# An Autobiographical Case Report of Eosinophilic Esophagitis: A Diagnosis That Is Hard to Swallow

**DOI:** 10.7759/cureus.38852

**Published:** 2023-05-10

**Authors:** Vincenzo Happach, Matthew Daniel, Jason Buckert

**Affiliations:** 1 Emergency Medicine, Piedmont Macon Medical Center, Macon, USA

**Keywords:** emergency medicine, gi, immunology, gastroenterology, esophagitis, eosinophilic esophagitis

## Abstract

This case is an autobiographical report and a first-hand account of my experience with eosinophilic esophagitis. It follows my realization of symptoms through food bolus obstruction, treatment with steroids and proton pump inhibitors, and remission of symptoms. This case shows how even someone with healthcare experience can go years without an appropriate diagnosis of this poorly understood condition.

## Introduction

The updated consensus recommendations published in the Journal of Allergy and Clinical Immunology define eosinophilic esophagitis (EoE) as a chronic esophageal disease that is likely allergy/immunology mediated and characterized clinically by esophageal dysfunction and histologically by eosinophil-predominant inflammation [1]. This definition is an update from the 2007 consensus that is rarely used by clinicians for the diagnosis of EoE [1]. EoE is most seen in males during the second and third decades of life. Studies have shown that food impaction is present in 54% of patients, and 15% have dysphagia [2]. This case explores a young physician’s experience through diagnosis, treatment, and maintenance of this condition.

## Case presentation

My parents recall me “always choking on something” as a young child. Looking back through my adolescent years, I remember frequently needing to drink while I ate to help “wash down” food. As a teenager and young adult, I can recall a few instances of the sensation of impaction causing me to retch and vomit until it would pass. The common culprits were steak, sausage, or most of all chicken wings. The food would eventually pass after some time. Therefore, I just figured I needed to chew my food better or take smaller bites. In 2013, I was a transitional year intern at the Naval Medical Center Portsmouth. During an emergency medicine shift, a fellow intern treated a patient presenting with a food bolus obstruction requiring evaluation by gastroenterology and ultimately endoscopic removal. This was when I finally realized I had been struggling with episodes of esophageal obstruction myself.

At that time in my life, I was unaware of the existence of EoE and thought I likely had some sort of physical obstruction such as an esophageal stricture or web. Two weeks later, I underwent an esophagogastroduodenoscopy (EGD) with mucosal biopsies and was found to have diffuse mild mucosal abnormalities including longitudinal markings and tight circumferential folds in the middle and lower thirds of the esophagus. Biopsies obtained were notable for eosinophilic infiltration of >200/hpf. With those results, my gastroenterologist diagnosed me with EoE and started me on a regimen of omeprazole 20mg daily and eight weeks of budesonide slurry by mouth. Over the next two months, my symptoms of occasional dysphagia resolved and have remained so on oral omeprazole daily. As PPIs alone resolved my symptoms, I did not require treatment with maintenance steroids. I have not had a recurrence of food bolus obstruction or dysphagia since the initiation of treatment seven years ago. In my career with the Navy, I embarked on duty involving flying which required a medical flight waiver for my diagnosis of EoE. Though I was not required to undergo repeat EGD as I was symptom-free, part of this waiver required evaluation by an allergist. I had a history of seasonal allergies but no known food allergies or atopy. I underwent skin patch testing that was notable for reactions to several weeds and grasses but without reaction to any common foods.

## Discussion

EoE is a poorly understood gastrointestinal condition in which inflammatory eosinophilic infiltration occurs in the mucosal lining of the esophagus. For many years, eosinophilic infiltration of the esophagus was considered a normal finding or related to gastroesophageal reflux disease (GERD). This leads to symptoms of dysphagia, GERD, and food bolus obstruction in adults [3].

**Figure 1:**
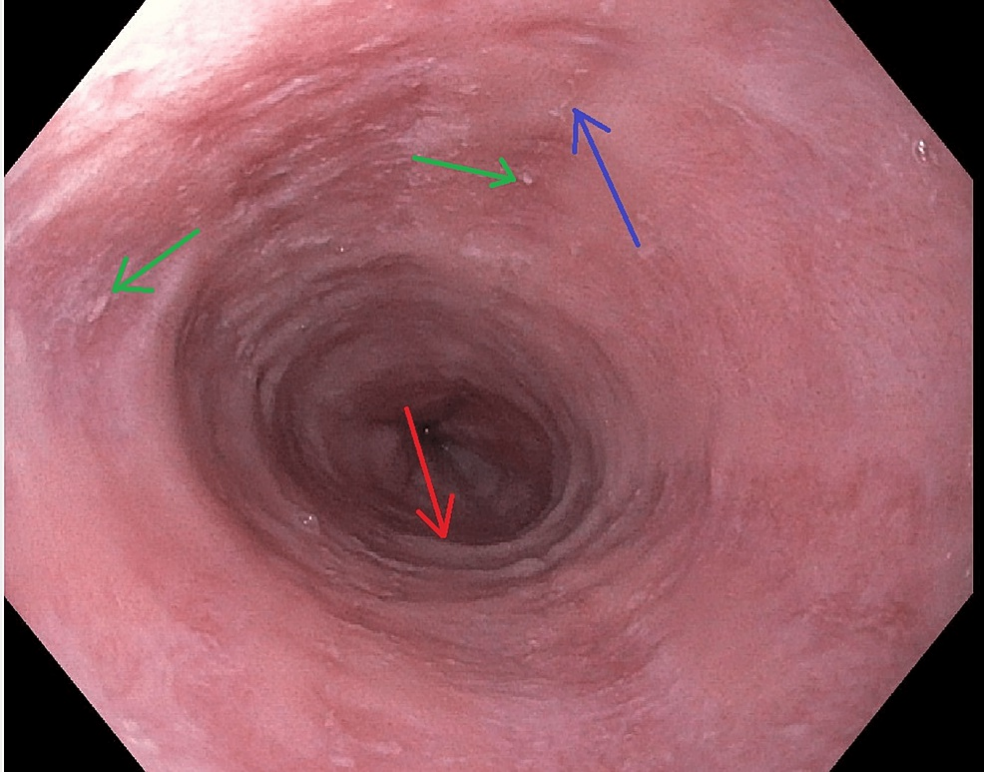
Upper endoscopy at the distal esophagus demonstrating diffuse edema, rings (red arrow), exudates (green arrows), and furrows (blue arrow)

**Figure 2:**
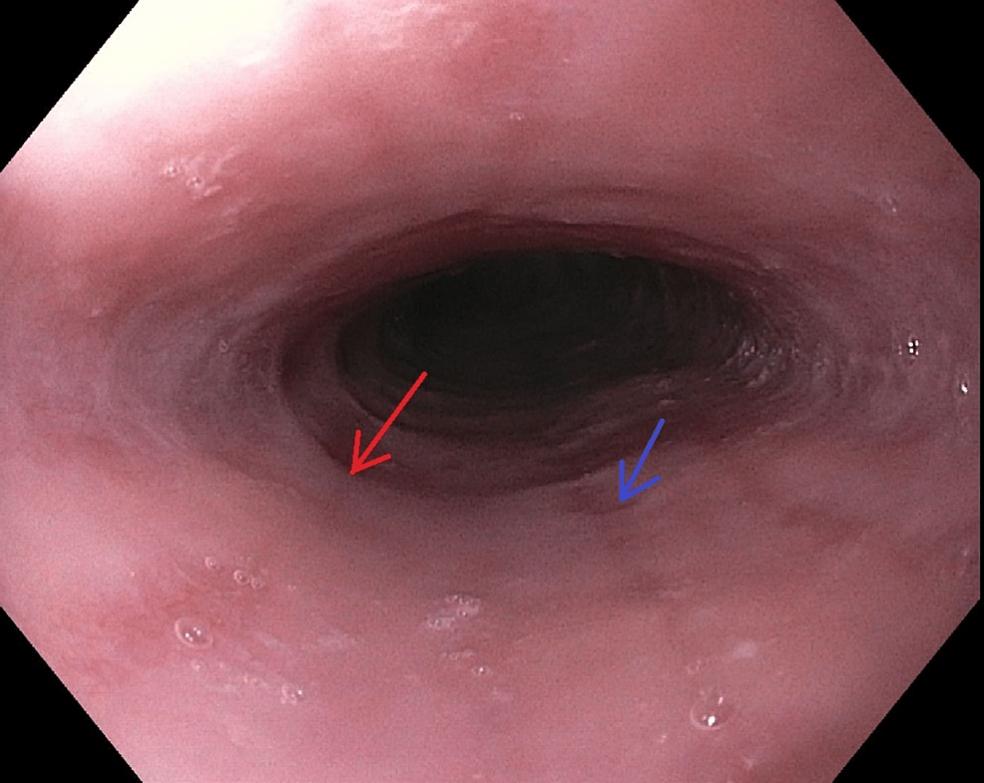
Upper endoscopy at the mid esophagus with examples of rings (red arrow) and furrows (blue arrow)

The exact trigger of EoE has been difficult to elucidate. Recent studies suggest that sensitization from food-specific IgE and T-helper lymphocyte type 2 cells appear to contribute to the pathogenesis along with basophils, mast cells, and other antigen-presenting cells [4]. These studies have been performed in animal models and reviews from patients over many years [4]. Diagnosis is often made by endoscopy with biopsy which will undergo histologic examination to look for eosinophilia. Official diagnosis requires all three of the following features: esophageal dysmotility or related symptoms, eosinophilic inflammation on biopsy with a peak value of >15 eosinophils per high-powered field, and exclusion of other causes [5]. Endoscopy is an excellent tool to not only diagnose EoE but also to rule out other etiologies of dysphagia [6]. Characteristic findings of EoE on endoscopic evaluation include circular rings, strictures, subepithelial vascular patterns, linear furrows, and white papules/spots suggesting eosinophilic abscesses [6]. Examples of circular rings, linear furrows, and eosinophilic exudates are demonstrated in Figures 1, 2.

There are multiple treatment modalities for EoE. Often, an elimination diet is the first course of action in an attempt to improve symptoms. Having patients keep a food log to determine triggering items and removing them from the diet can sometimes alone be effective in resolving the symptom. Unfortunately, elimination diets are often ineffective or unsustainable due to patient motivation, resources, and lifestyle. Acid suppression with PPI therapy is another treatment modality found to be effective. It is particularly effective in patients with EoE secondary to GERD or a yet-to-be-defied PPI-responsive EoE [1]. Patients with EoE that is not caused by GERD may have a poor response to acid suppression and will require further treatment. Corticosteroids can be used to induce remission of symptoms of EoE, as it was used in the case described. It also improves the pathologic features of EoE but most recur after discontinuation [1]. Currently, it’s not recommended for prolonged use due to concerns about adrenal suppression and its effects on bone density [1]. The role of esophageal dilatation in cases of stricture formation as a result of EoE is controversial. In initial studies, it appeared esophageal dilatation in EoE patients who had a higher complication rate than those with other benign strictures [1]. More recent studies push against these initial findings. The current recommendation is that a more conservative approach be taken with EoE patients when pared to those with benign strictures, such as using multiple sessions of gradual dilatation [1].

## Conclusions

With my diagnosis, there was no clear food-specific allergen I could attribute to my symptoms, and antihistamine usage does not appear to make a difference in my symptoms. I am fortunate in that my disease process is amendable to daily PPI therapy without recurrence of symptoms. Subsequent EGD performed have shown resolution of exudates, furrows, and rings, but biopsies still show some mild eosinophilic infiltration, but it is below the threshold for active disease. Since my diagnosis, I continue to live a symptom-free life while only requiring 20 mg of omeprazole a day. I’ve attempted vacations from and lower doses of PPI, but after 4-5 days, minor dysphagia would begin returning with meals. That said, I take my meds and enjoy a normal diet, including chicken wings.
